# Electrocatalytic Oxidation as an Effective Pretreatment for Ultrafiltration Fouling Control: Modulation of EfOM Characteristics and Fouling Pathways

**DOI:** 10.3390/membranes16100317

**Published:** 2026-09-25

**Authors:** Tao Xu

**Affiliations:** PipeChina Institute of Science and Technology, Tianjin 300450, China; xutao62@pipechina.com.cn

**Keywords:** electro-oxidation pretreatment, ultrafiltration membrane, membrane fouling, effluent organic matter

## Abstract

Ultrafiltration membranes are widely used in wastewater reuse systems, but membrane fouling caused by effluent organic matter (EfOM) remains a major limitation. In this study, electrocatalytic oxidation (EO) was applied as a pretreatment strategy to regulate EfOM properties and alleviate UF membrane fouling. The effects of EO pretreatment at different current densities on membrane performance, organic matter transformation, and fouling mechanisms were systematically investigated. The results demonstrated that EO significantly enhanced flux stability and fouling reversibility with different current densities. These improvements were associated with changes in EfOM characteristics after EO treatment, including decreased DOC and UV_254_ values, a shift toward lower-molecular-weight fractions, and variations in fluorescent organic components. Fouling model analysis revealed that EO pretreatment shifted the dominant fouling mechanism from cake layer formation toward pore-blocking process. This study provides insights into EO-assisted fouling control and highlights its potential as an effective pretreatment approach for improving UF performance.

## 1. Introduction

Ultrafiltration (UF) has become an important technology for water treatment because of its high efficiency in removing suspended solids, colloids, microorganisms, and dissolved organic matter from wastewater [1,2,3]. However, membrane fouling remains one of the major challenges limiting long-term operation, leading to flux decline, shortened membrane lifespan, and increased operational and maintenance costs [4,5]. Among various foulants, effluent organic matter (EfOM) is considered one of the major contributors to UF membrane fouling owing to its compositional complexity and heterogeneous physicochemical properties [6,7,8]. Therefore, developing effective pretreatment strategies to mitigate membrane fouling associated with EfOM is important for improving UF performance [9,10].

Among the available pretreatment approaches, advanced oxidation processes (AOPs) have attracted considerable attention because they can transform or partially mineralize organic foulants before membrane filtration, thereby alleviating membrane fouling and improving permeate flux [11,12,13]. Conventional AOPs based on ozone (O_3_), chlorine dioxide (ClO_2_), and hydrogen peroxide (H_2_O_2_) have demonstrated promising performance in fouling mitigation [14,15]. Ozone oxidizes organic contaminants either directly or indirectly through the generation of reactive oxygen species (ROS) during its decomposition [16]. However, its practical application is constrained by its low aqueous solubility and poor stability [17]. Moreover, ozone often induces partial oxidation rather than complete mineralization of complex organic matter such as EfOM [18]. Chlorine dioxide has also been reported to mitigate membrane fouling by weakening foulant–membrane interactions. Nevertheless, its effectiveness in suppressing irreversible membrane fouling remains limited [12]. Electrocatalytic oxidation (EO) has recently emerged as an advanced oxidation technology owing to its ability to generate highly reactive oxidizing species in situ without the addition of chemical oxidants [19]. Under an applied electric field, reactive oxygen species, including hydroxyl radicals (•OH), superoxide radicals (O_2_^•−^), and other oxidative intermediates, are continuously produced on the electrode surface, enabling efficient degradation and transformation of organic contaminants [20]. However, several important questions remain unclear regarding the application of EO as a UF pretreatment. Specifically, it remains unclear how EO modifies the physicochemical characteristics of EfOM and to what extent EO pretreatment can mitigate reversible and irreversible membrane fouling. Addressing these questions is important for elucidating the mechanisms involved in EO-assisted fouling control.

In this study, we systematically investigated electrocatalytic oxidation as a pretreatment strategy to alleviate membrane fouling associated with EfOM during ultrafiltration. The changes in EfOM characteristics, including molecular weight distribution, functional group composition, and aggregation behavior, are comprehensively analyzed to clarify their effects on foulant–membrane interactions. Meanwhile, the resulting impacts on both reversible and irreversible fouling resistance are quantitatively evaluated under varying EO operating conditions, and the membrane fouling mechanisms were explored. This study provides new insights into the interfacial processes linking electrochemical oxidation and membrane filtration and indicates the potential application of EO as a pretreatment approach for enhancing UF performance.

## 2. Materials and Methods

### 2.1. Experimental Materials

All the materials used were of analytical grade. Sodium hydroxide (NaOH), hydrochloric acid (HCl), and sodium chloride (NaCl) were purchased from the Sinopharm Chemical Reagent, China. UF membranes made of polyethersulfone (PES-10K, Mosu, Shanghai, China) were employed (effective filtration area, 50 cm^2^; molecular weight cut-off, 10 kDa; c; contact angle, 60.3 ± 0.5). The electrodes were purchased from Shanxi Baigong Jiahe Titanium Industry Co., Ltd., BaoJi, China. The water samples were collected from the effluent of the secondary sedimentation tank of a municipal wastewater treatment plant (MWTP) located in northeastern China. The dissolved organic carbon (DOC), UV_254_, and pH of the raw water were 36.5 ± 2 mg/L, 0.185 ± 0.025 cm^−1^, and 7.8 ± 0.2, respectively. All samples were stored at 4 ± 1 °C to minimize changes in their physicochemical properties.

### 2.2. Batch Experiments

The schematic of the EO system coupled with the ultrafiltration process is shown in Figure 1. In a typical experiment, the feed water was first introduced into the electrochemical oxidation reactor, where it was treated for 0.5 h. A boron-doped diamond (BDD) electrode (4 cm × 5 cm) was used as the anode, stainless-steel electrodes as a cathode, and the applied current densities were set at 20, 50, and 70 mA cm^−2^, respectively. For the UF system, a polyethersulfone (PES) membrane with an effective filtration area of 50 cm^2^ and a molecular weight cut-off of 10 kDa was used. Prior to use, each new membrane was immersed in ultrapure water for 12 h to ensure stable initial permeability. The membrane was then installed at the bottom of a dead-end ultrafiltration cell (Mosu, Shanghai, China). Filtration was conducted under a constant transmembrane pressure of 0.1 MPa, maintained by nitrogen gas. During operation, the permeate was continuously collected, and its mass was recorded using an electronic balance connected to a computer for real-time data acquisition. During the backwash process, the used membrane was reversed, and an elevated pressure of 0.2 MPa was applied to the permeate side.

### 2.3. Membrane Fouling Analysis

The resistance-in-series model was applied to evaluate the effects of EO pretreatment on membrane fouling characteristics by using Darcy’s Law. The model can be expressed as follows:

(1) $$J=\frac{∆P}{\mu R_{t}}$$

(2) $$R_{t}=R_{m}+R_{r}+R_{ir}$$

where J is the permeation flux (m/s); ∆P represents the transmembrane pressure (Pa); μ is the dynamic viscosity (Pa s); R_t_ is the total hydraulic membrane resistance; R_m_ is the intrinsic resistance of the clean membrane (m^−1^); R_r_ is the hydraulic reversible membrane resistance (m^−1^); R_ir_ represents hydraulic irreversible membrane resistance (m^−1^).

Ultrapure water filtration (300 mL) was performed before each experiment, and the average permeate flux was designated J_0_. R_m_ could be calculated using Equation (3).

(3) $$R_{m}=\frac{∆P}{\mu J_{0}}$$

To calculate the R_t_, pretreatment water sample filtration (300 mL) was performed, and the final permeate flux was designated J_1_. R_t_ could be calculated using Equation (4).

(4) $$R_{t}=\frac{∆P}{\mu J_{1}}$$

The fouled membrane was backwashed via inverting the membrane in the reactor and filtrating 30 mL of ultrapure water. Then, the backwashed membrane was placed in its original position followed by 100 mL of ultrapure water filtration. The average permeate flux was designated as J_2_. Additionally, R_r_ and R_ir_ were calculated by Equations (5) and (6), respectively [7].

(5) $$R_{r}=\frac{∆P}{\mu J_{1}}-\frac{∆P}{\mu J_{2}}$$

(6) $$R_{ir}=R_{t}-R_{m}-R_{r}$$

The classic fouling models, namely complete blocking, standard blocking, intermediate blocking, and cake filtration, were used to better understand the membrane fouling mechanism [21].

Complete blocking could be calculated using Equation (7).

(7) $$R_{r}-J=A\times V$$

Standard blocking could be calculated using Equation (8).

(8) $$\frac{1}{t}+B=\frac{J_{0}}{V}$$

Intermediate blocking could be calculated using Equation (9).

(9) $$InJ_{0}-InJ=C\times V$$

Cake blocking could be calculated using Equation (10).

(10) $$(\frac{1}{J})-(\frac{1}{J_{0}})=D\times V$$

In the equations above, V is the accumulative volume, t is the filtration time, and A, B, C and D are constants, respectively.

### 2.4. Analytical Methods

The pH of the samples was measured using a pH meter (PHS-3C, Leici, Shanghai, China). DOC was determined with a total organic carbon (TOC) analyzer (N-C3100, Analytik Jena AG, Freistaat Thüringen, Germany). UV absorbance at 254 nm (UV_254_) was measured using a UV–visible spectrophotometer (DR5000, Hach Company, Loveland, CO, USA) with a 1 cm path length quartz cell. The excitation–emission matrix (EEM) was measured with a fluorescence spectrophotometer (F7000, Hitachi High-Tech Science Corporation, Hitachi, Japan) using a quartz cuvette to characterize fluorescent components in the water samples. The EEM of Milli-Q water was subtracted from each EEM sample to eliminate Raman scattering signals. The molecular weight distribution of dissolved organic matter in the secondary effluent was determined using an ultrafiltration fractionation method. Membranes with molecular weight cut-offs of 50, 10, 5, and 1 kDa were sequentially applied, and the DOC concentrations of the corresponding permeates were measured to quantify each fraction. After filtration experiments, fouled membranes were carefully retrieved and rinsed with deionized water to remove loosely attached surface deposits. The surface morphology of both pristine and fouled membranes was characterized using scanning electron microscopy (SEM; SU8010N, Hitachi High-Tech Corporation, Tokyo, Japan). In addition, chemical functional groups on the membrane surfaces were analyzed by Fourier transform infrared spectroscopy (FTIR; Thermo Fisher Scientific, Waltham, MA, USA) to elucidate the nature of membrane foulants.

The three-dimensional fluorescence spectrum can be quantified by the fluorescence regional integration (FRI) method. The excitation wavelength (EX) and emission wavelength (EM) were divided into regions of 5 nm × 5 nm, with each small area having its own fluorescence intensity value. The fluorescence response of each region was calculated as follows:

(11) $$\begin{array}{l} \Phi_{i,n}=M\\ F_{i}\int_{ex}\int_{em}I(\lambda_{ex}\lambda_{em})d\lambda_{ex}d\lambda_{em} \end{array}$$

(12) $$P_{i,n}=\frac{\Phi_{i,n}}{\Phi_{T,n}}\times 100\%$$

where $\Phi_{i,n}$ reflects the cumulative fluorescence response of organic matter at Region I; I ($\lambda_{ex}\lambda_{em}$) is the fluorescence intensity at each EX-EM wavelength; M$F_{i}$ is the ratio of the total area to each EX-EM area; $\Phi_{T,n}$ is the sum of $\Phi_{i,n}$; $P_{i,n}$ is the percent of fluorescence response in Region I.

## 3. Results and Discussion

### 3.1. Effect of EO Pretreatment on Membrane Filtration Performance

The effects of EO pretreatment on specific flux and fouling reversibility during ultrafiltration under a transmembrane pressure of 0.1 MPa are shown in Figure 2. For untreated feedwater, the specific flux rapidly decreased during the first filtration cycle, reaching 0.80 of the initial flux value at the end of the cycle (Figure 2a). Although backwashing partially restored the flux to 0.84, the specific flux continued to decline during subsequent filtration cycles, reaching 0.72 after three cycles. These results suggest that backwashing removed loosely attached foulants, whereas residual fouling resistance accumulated during repeated filtration cycles. These results were consistent with the fouling resistance analysis (Figure 2b), where untreated feedwater exhibited the highest fouling resistance. The total and irreversible fouling resistances reached 2.35 × 10^11^ m^−1^ and 1.85 × 10^11^ m^−1^, respectively, indicating substantial accumulation of irreversible fouling during filtration.

In contrast, EO pretreatment reduced flux decline and improved flux recovery after backwashing. At a current density of 20 mA cm^−2^, the specific flux reached 0.84 at the end of the first cycle and further increased to 0.88 after backwashing. Correspondingly, the total and irreversible fouling resistances decreased to 1.85 × 10^11^ m^−1^ and 1.31 × 10^11^ m^−1^, respectively. At 50 and 70 mA cm^−2^, the specific flux values after the first filtration cycle were 0.85 and 0.87, respectively, and increased to 0.89 after backwashing. After three filtration cycles, the final fluxes remained relatively stable at 0.85 and 0.88. At 70 mA cm^−2^, reversible and irreversible fouling resistances decreased by approximately 75% and 30%, respectively, compared with untreated feedwater. These results indicate that EO pretreatment can reduce flux decline and alleviate both reversible and irreversible fouling during ultrafiltration cycles. The improved flux recovery suggests that EO treatment modifies EfOM properties and reduces their tendency to form persistent deposits on the membrane surface.

### 3.2. Transformation of Dissolved Organic Matter by EO Pretreatment

DOC and UV_254_ were used to evaluate changes in effluent organic matter after EO pretreatment. As shown in Figure 3, UF alone showed limited removal of organic matter, with DOC and UV_254_ reductions of 4.8% and 8.3%, respectively. EO pretreatment enhanced the removal of organic compounds under different current densities. DOC removal of feedwater increased to 31.2%, 44.5%, and 63.5% at current densities of 20, 50, and 70 mA cm^−2^, respectively. A similar trend was observed for UV_254_, which increased to 58.4%, 77.5%, and 82.3% under the same conditions. These results are consistent with the findings of Zeng et al., who reported that oxidative pretreatment achieving 64% DOC and 76% UV_254_ removal also resulted in substantial mitigation of membrane fouling [22]. The higher removal of UV_254_ compared with DOC suggests that EO preferentially transformed chromophoric and aromatic structures, particularly unsaturated and conjugated compounds. These results suggest that EO treatment altered EfOM composition, which may reduce the tendency of organic compounds to contribute to membrane fouling during subsequent ultrafiltration.

Analysis of molecular weight (MW) distribution (Figure 4) revealed that EO pretreatment gradually shifted the distribution of organic matter from high-molecular-weight fractions toward lower-molecular-weight fractions. In untreated feedwater, the dominant fractions were 50–10 kDa and 10–5 kDa, accounting for 35.9% and 27.4%, respectively. Following EO pretreatment, the distribution shifted toward smaller molecular weight fractions. At a current density of 20 mA cm^−2^, the proportion of the 50–10 kDa fraction decreased to 23.1%, accompanied by an increase in lower-molecular-weight fractions. Increasing the current density to 50 mA cm^−2^ further promoted the shift toward the 10–5 kDa and 5–1 kDa fractions. At 70 mA cm^−2^, the 5–1 kDa fraction became dominant, accounting for 65.9% of total organic matter. These results suggest that EO promoted the oxidative transformation of high-molecular-weight organic compounds into smaller molecular fractions.

Changes in EfOM fluorescent characteristics after electrocatalytic oxidation pretreatment were further investigated using excitation–emission matrix (EEM) fluorescence spectroscopy combined with fluorescence regional integration (FRI) analysis (Figure 5 and Table 1). As shown in the EEM profiles, untreated water exhibited fluorescence signals associated with protein-like (Regions I and II) and humic/fulvic-like components (Region III), indicating the presence of different fluorescent organic fractions. Following EO pretreatment, fluorescence intensity decreased across most excitation–emission regions. At 20 mA cm^−2^, the fluorescence intensity of humic/fulvic-like components decreased, accompanied by changes in the relative contribution of protein-like fluorescence regions. With increasing current density, this trend became progressively more pronounced, and at 70 mA cm^−2^ nearly all humic-like fluorescence signals were substantially suppressed, indicating extensive degradation of aromatic structures.

FRI analysis further quantified the changes in fluorescence distribution. In untreated water, the fluorescence of protein-like (Region I) and tryptophan-like (Region II) substances accounted for 43.8% and 41.6% of the total fluorescence response, respectively, while humic-like substances (Region III) contributed 14.6%. After EO pretreatment, the relative contributions of different fluorescence regions changed, with Region II decreasing to 16.0–19.2% and Region III increasing to 29.4–31.9%. This variation may result from the preferential transformation of certain fluorescent components during EO treatment. Meanwhile, Region I remained relatively stable or slightly increased (~51–52%), suggesting that protein-like fluorescent components were less affected compared with other fluorescence fractions. These fluorescence results were consistent with the DOC and UV_254_ measurements, which showed reductions in organic carbon and UV_254_ absorbance of up to 63.5% and 82.3%, respectively, at the highest current density.

In addition, the effects of EO pretreatment on membrane foulant deposition and the chemical characteristics of deposited foulants were further investigated using SEM and FTIR analyses. SEM images showed that untreated feedwater resulted in the formation of a relatively dense fouling layer on the membrane surface, whereas EO pretreatment reduced the amount of deposited material and resulted in a less compact surface morphology with increasing current density (Figure 6). FTIR spectra further revealed changes in the characteristic functional groups of deposited foulants after EO pretreatment. The decreased intensities of peaks associated with O–H, C–O, and C–O–C groups suggested alterations in the chemical composition of membrane foulants after oxidation treatment (Figure 7). We further compared the newly obtained SEM images and FTIR spectra of the membranes before fouling, after fouling, and after backwashing. The results also indicate that EO pretreatment reduced the deposition of EfOM-derived foulants on the membrane surface (Figures S5 and S6).

### 3.3. The Models of Membrane Fouling

Membrane fouling behavior was further analyzed using classical filtration models, including complete blocking, standard blocking, intermediate blocking, and cake filtration models. The corresponding fitting results are summarized in Table 2 and Figures S1–S4. For untreated feedwater, all four models showed relatively high correlation coefficients. The cake filtration model exhibited the highest fitting value (R^2^ = 0.987), followed by the intermediate blocking models (R^2^ = 0.961) and standard blocking (R^2^ = 0.915). These results suggest that surface deposition contributed substantially to fouling development under untreated conditions. After EO pretreatment, changes in model fitting behavior were observed. The R^2^ values of the standard blocking model increased 0.940 and 0.998 at 50 mA cm^−2^ and 70 mA cm^−2^, respectively. In contrast, the fitting coefficient of the cake filtration model decreased from 0.962 at 20 mA cm^−2^ to 0.916 at 70 mA cm^−2^, suggesting a reduced contribution of cake-layer-associated fouling behavior after EO pretreatment. A similar decreasing trend was observed for the complete blocking model, whereas the intermediate blocking model remained relatively stable with slightly lower fitting values than the untreated system. Overall, these results suggest that EO pretreatment altered the fouling behavior during ultrafiltration by reducing the contribution of severe surface deposition processes.

### 3.4. Membrane Fouling Behavior and Mechanistic Interpretation

The results from flux evolution, fouling resistance analysis, fouling model fitting, and membrane surface characterization suggest that EO pretreatment substantially influences membrane fouling behavior during ultrafiltration. Without EO pretreatment, membrane fouling exhibited characteristics associated with multiple filtration mechanisms, with the cake filtration model showing the highest fitting performance among the evaluated models. The higher fitting coefficient of the cake filtration model suggests that surface deposition may contribute substantially to resistance development under untreated conditions. The presence of high-molecular-weight organic fractions may facilitate the formation of surface deposits during filtration. This behavior is consistent with the presence of relatively high proportions of protein-like and humic/fulvic-like fluorescent components, which may contribute to foulant accumulation and increased filtration resistance. Following EO pretreatment, changes in fouling model fitting behavior were observed. With increasing current density, the contribution of the cake filtration model decreased, whereas the standard blocking model showed improved fitting performance. The lower fitting coefficients of intermediate and complete blocking models further suggest changes in the contribution of different fouling processes after EO treatment. These changes in fouling behavior were associated with alterations in dissolved organic matter properties after EO treatment, including redistribution of molecular weight fractions toward lower-molecular-weight compounds, changes in fluorescence characteristics, and reductions in DOC and UV_254_ absorbance. SEM and FTIR analyses further indicated changes in foulant deposition morphology and chemical characteristics after EO pretreatment. Overall, EO pretreatment reduced membrane fouling severity by modifying EfOM characteristics and decreasing the formation of strongly deposited foulants. The resulting fouling behavior showed a lower contribution from cake-layer-associated filtration resistance, which contributed to improved UF performance.

In addition, we calculated the difference in filtration costs caused in UF and UF + EO systems (Figure S7). The energy demand of EO+ UF was 1.8, 3.2, and 4.7 times higher than UF systems. Although increasing current density enhanced EfOM degradation and membrane fouling control, the corresponding energy demand also increased. Notably, the additional fouling mitigation at the highest current density did not increase proportionally with energy demand. Therefore, 50 mA cm^−2^ provided a favorable balance between fouling mitigation and energy demand and was selected as the optimum operating condition.

## 4. Conclusions

In this study, electrocatalytic oxidation was investigated as a pretreatment strategy for mitigating ultrafiltration membrane fouling during the treatment of municipal secondary effluent. EO pretreatment improved flux stability and recovery after backwashing, while reducing reversible and irreversible fouling resistances by approximately 75% and 30% at 70 mA cm^−2^. These improvements were associated with EO-induced transformations of dissolved organic matter, including reductions in DOC and UV_254_ absorbance, decreased proportions of high-molecular-weight fractions, and changes in fluorescent organic components, which collectively reduced the fouling potential of EfOM. Fouling analyses indicated reduced fouling resistance and changes in filtration model fitting behavior after EO pretreatment, suggesting a lower contribution of cake-layer-associated fouling processes. Overall, EO pretreatment modified EfOM characteristics and alleviated membrane fouling, indicating its potential application for improving UF performance in wastewater treatment. Nevertheless, further pilot-scale and long-term studies are needed to evaluate the energy consumption, electrode stability, by-product formation, and economic feasibility of the EO-UF process under continuous-flow operating conditions.

## Figures and Tables

**Figure 1:**
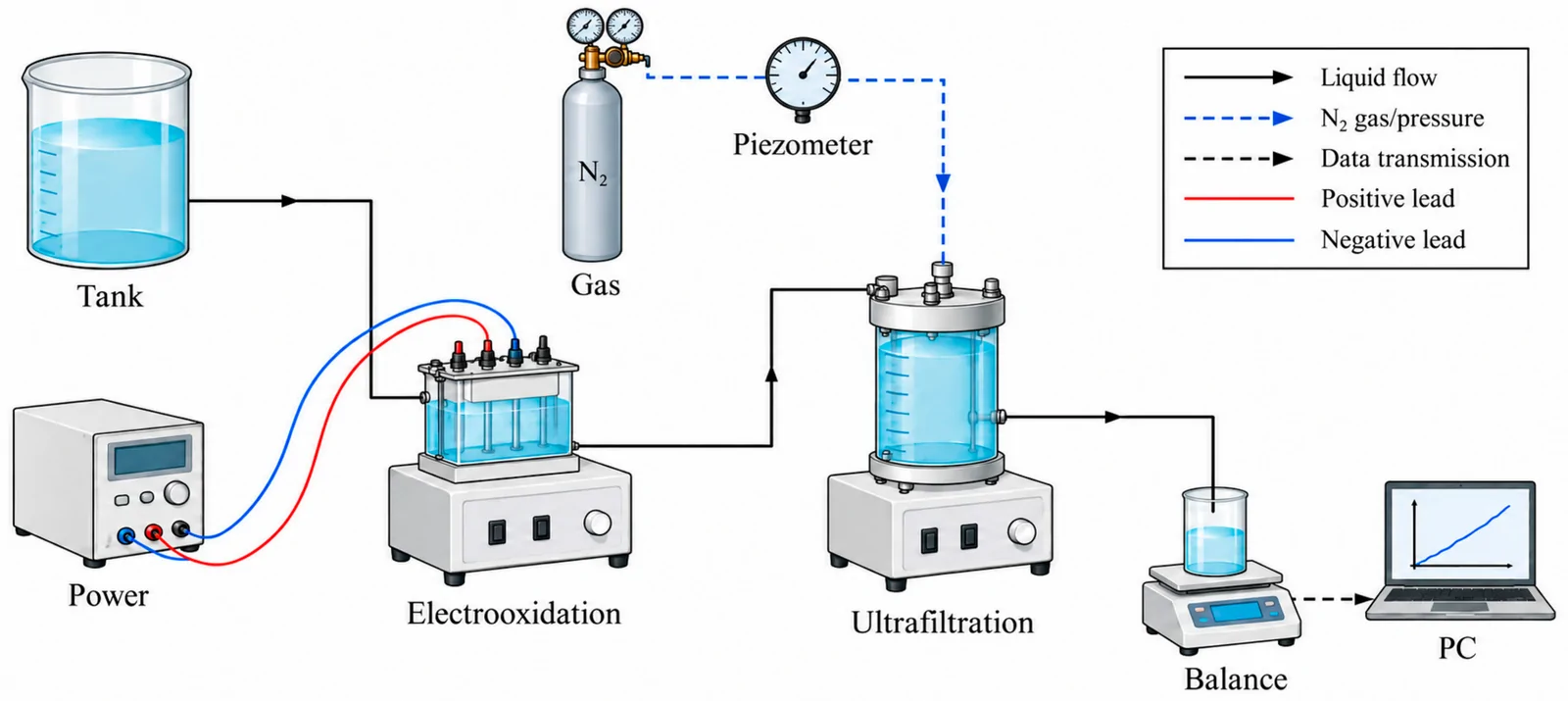
Schematic diagram of the EO-UF process.

**Figure 2:**
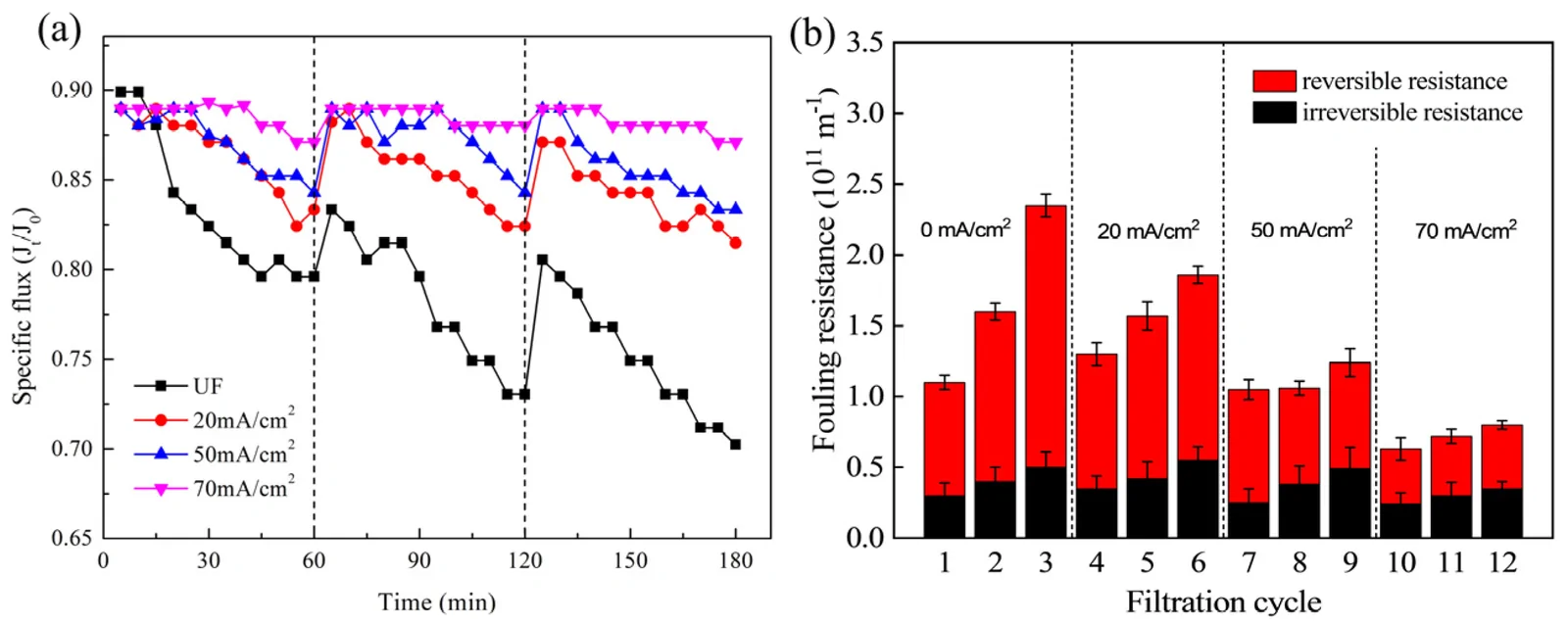
(**a**) Effects of EO pretreatment on specific flux decline with different current densities. (**b**) Effects of EO pretreatment on hydraulic reversible and irreversible resistance with different current densities.

**Figure 3:**
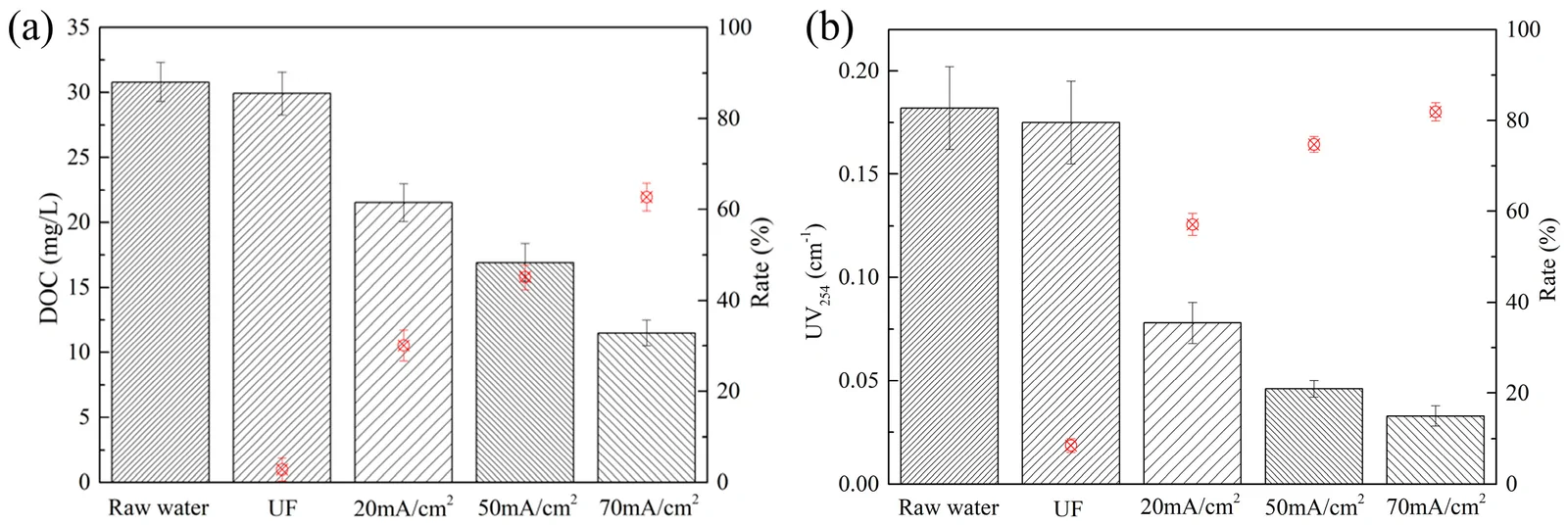
(**a**) Effect of EO pretreatment on DOC and (**b**) UV_254_ with different current densities. The red open-circle markers represent the removal rates, corresponding to the right-hand y-axis.

**Figure 4:**
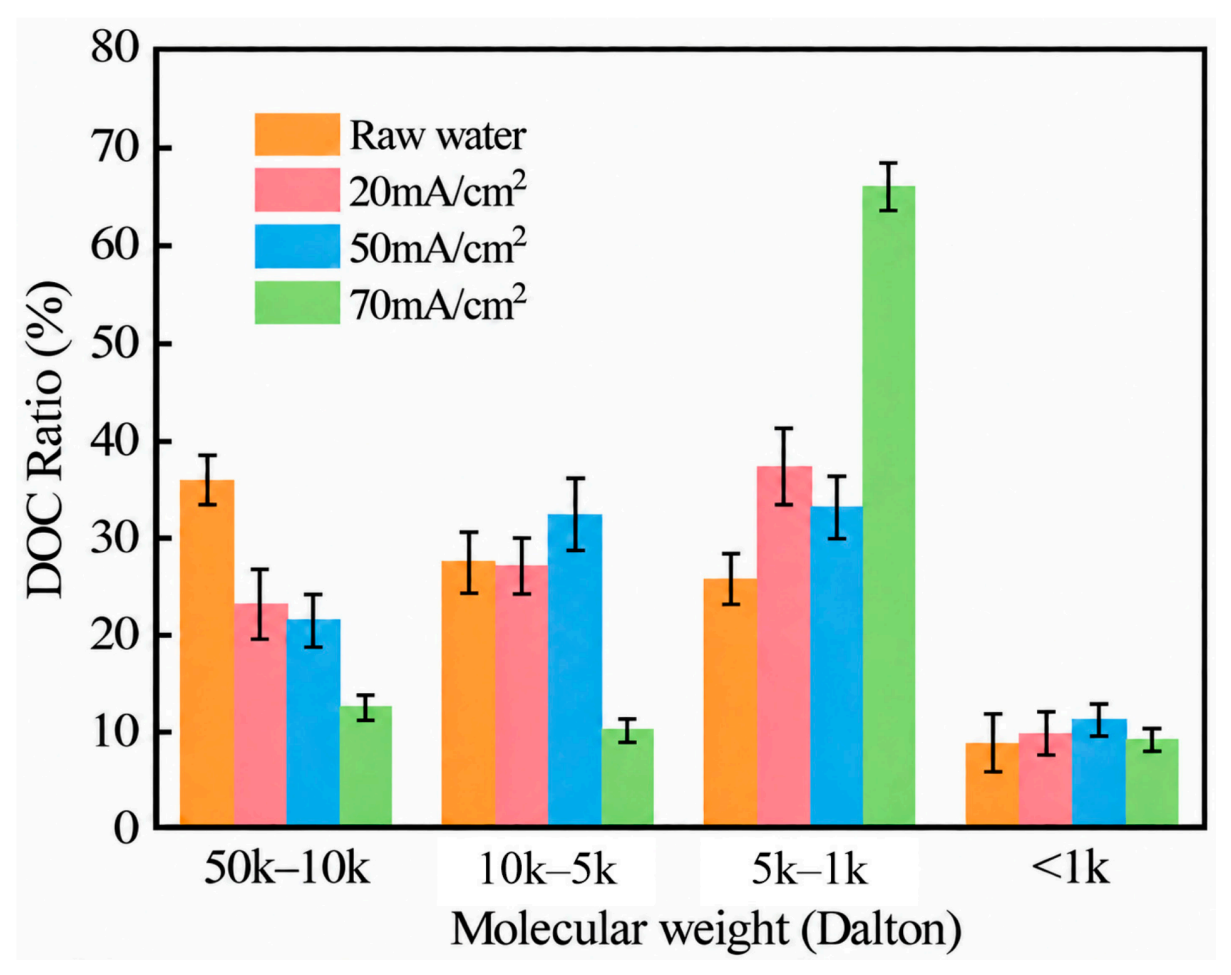
AMW composition of EfOM with different current densities.

**Figure 5:**
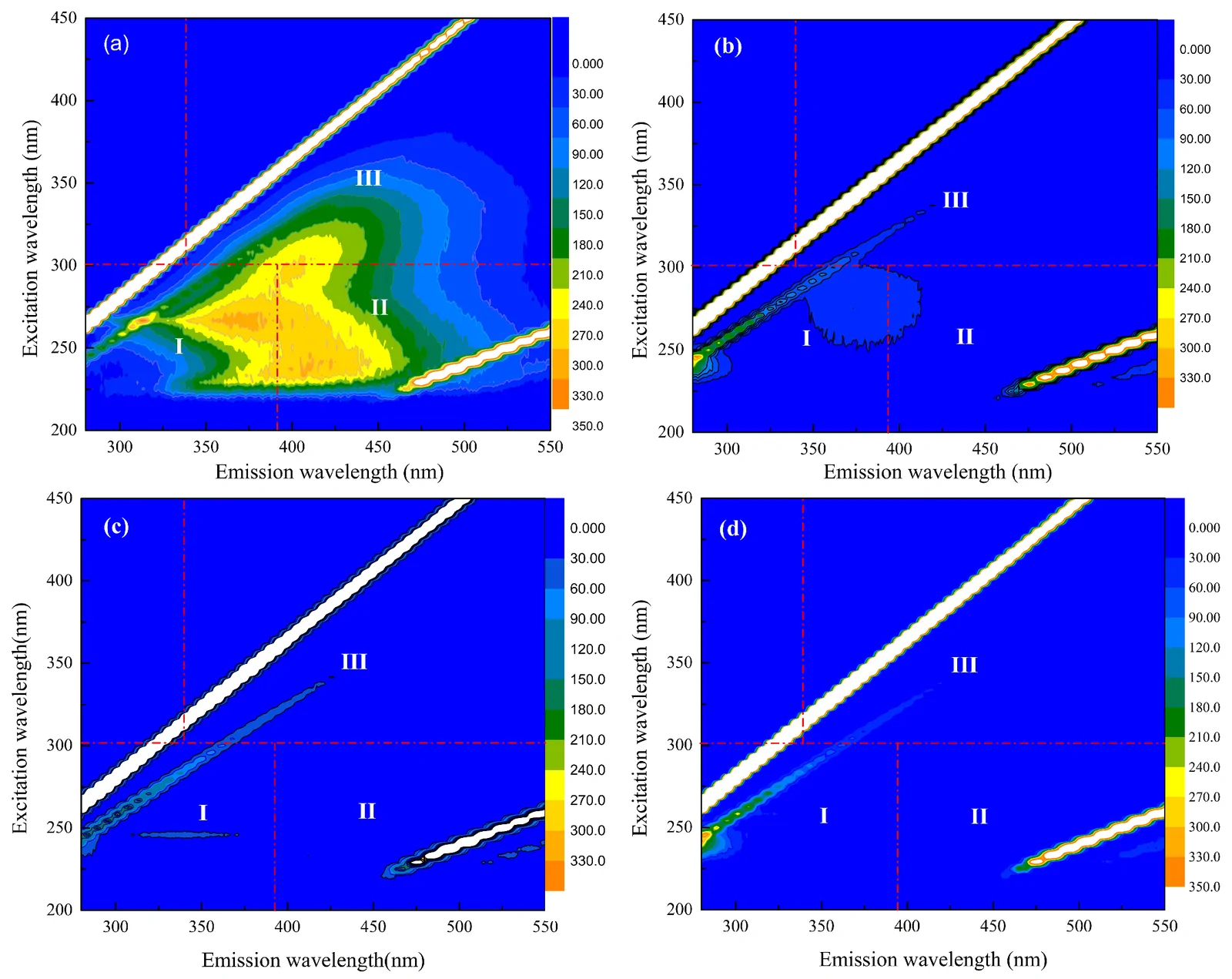
EEM fluorescence spectra of EfOM under different EO pretreatment conditions: (**a**) raw water sample; (**b**) EO pretreatment at 20 mA/cm^2^; (**c**) EO pretreatment at 50 mA/cm^2^; (**d**) EO pretreatment at 70 mA/cm^2^. Regions I, II, and III represent protein-like, fulvic acid-like, and humic acid-like fluorescent components, respectively.

**Figure 6:**
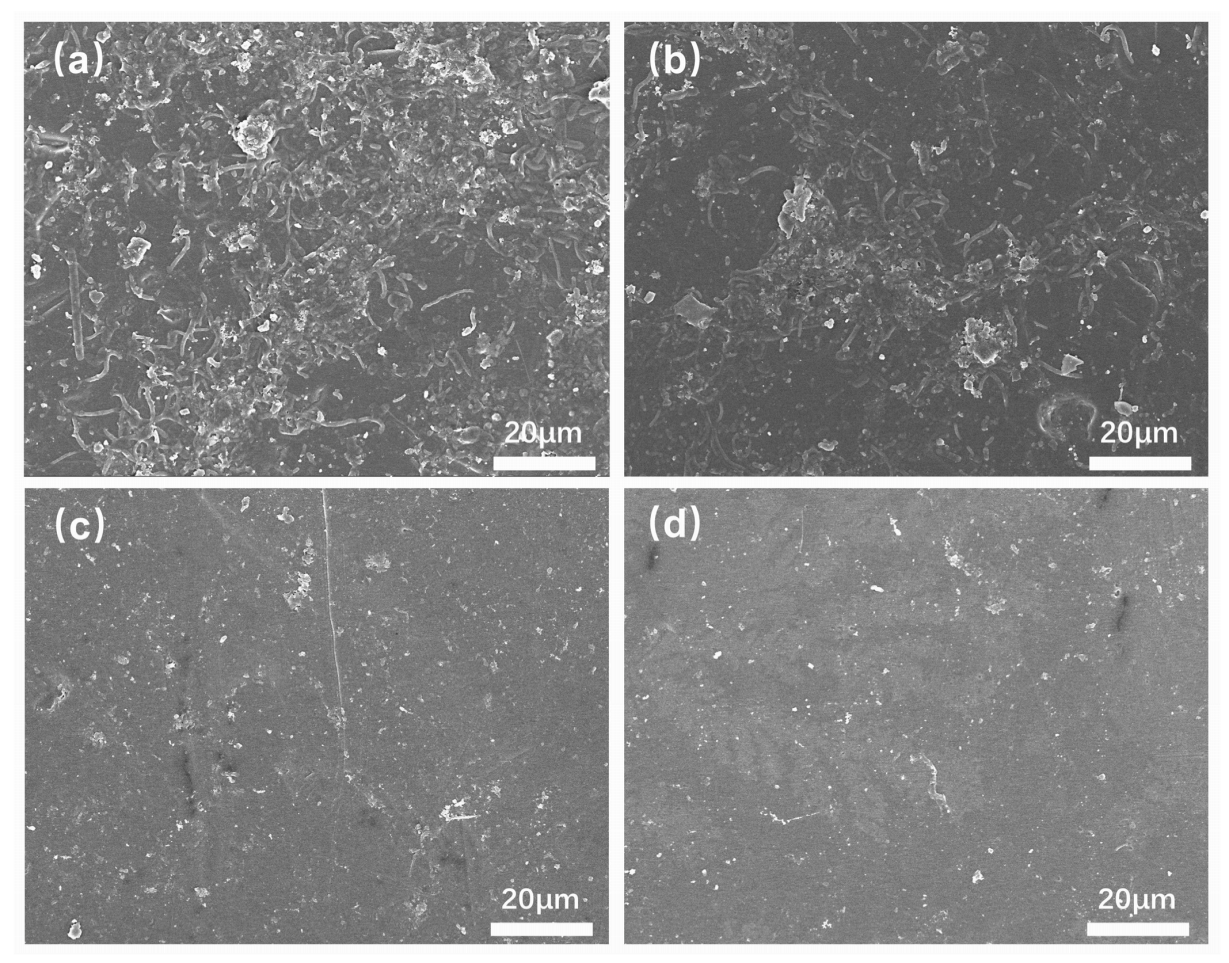
SEM images of fouled membranes under different pretreatment conditions: (**a**) without pretreatment; (**b**) EO pretreatment at 20 mA cm^−2^; (**c**) EO pretreatment at 50 mA cm^−2^; (**d**) EO pretreatment at 70 mA cm^−2^.

**Figure 7:**
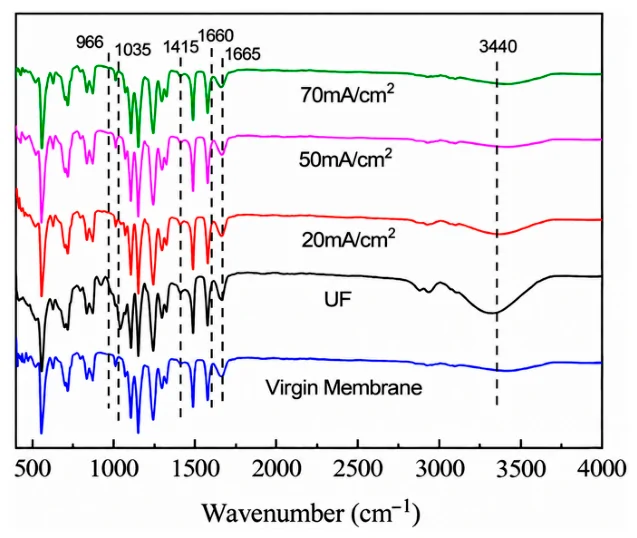
FTIR spectra of the fouled membrane by EO pretreatment with different conditions.

**Table 1:** FRI parameters for operationally defined EEM regions and EEM analysis of the effluents for EO pretreatment.

FRI Parameters			EEM Analysis							
EEM Region	Projected Excitation-Emission Area (nm^2^)	MFi	Raw Water		20 mA/cm^2^		50 mA/cm^2^		70 mA/cm^2^	
			$\Phi_{i,n}$ (×10^6^)	Pi,n (%)	$\Phi_{i,n}$ (×10^6^)	Pi,n (%)	$\Phi_{i,n}$ (×10^6^)	Pi,n (%)	$\Phi_{i,n}$ (×10^6^)	Pi,n (%)
I	7700	9.74	13.8	43.8	4.34	52.4	4.80	51.4	5.69	52.1
II	11,200	6.70	13.1	41.6	1.28	15.5	1.79	19.2	1.75	16.0
III	45,000	1.67	4.6	14.6	2.65	32.1	2.75	29.4	3.48	31.9
IV (Summation)	63,900		31.6	100	8.27	100	9.34	100	10.93	100

**Table 2:** R^2^ values of regression analyses for membrane fouling.

Feedwater	Complete Blocking	Standard Blocking	Intermediate Blocking	Cake Filtration
Raw water	0.867	0.915	0.961	0.987
20 mA/cm^2^	0.851	0.914	0.931	0.962
50 mA/cm^2^	0.814	0.940	0.941	0.951
70 mA/cm^2^	0.801	0.998	0.971	0.916

## Data Availability

All data generated or analyzed during this study are included in this published article and its Supplementary Information Files.

## Supplementary Materials

The following supporting information can be downloaded at: https://www.mdpi.com/article/10.3390/membranes16100317/s1.

## References

[1] Guo W., Ngo H.-H., Li J. (2012). A mini-review on membrane fouling. Bioresour. Technol..

[2] Liang Y.Y., Li M. (2026). Microplastic removal using membrane technologies: Challenges, fouling mitigation, and emerging AI-enabled solutions. Chem. Eng. Res. Des..

[3] Esenli B., Keskin B., Eryildiz-Yesir B., Al-Shaeli M., Irani-nezhad M.H., Khataee A., Koyuncu I., Vatanpour V. (2026). Enhancing the Performance of PVC Ultrafiltration Membranes Using Biotite and Biotene: Antifouling Properties and Separation Efficiency in Removal of Organic Pollutants. J. Appl. Polym. Sci..

[4] Peters C.D., Rantissi T., Gitis V., Hankins N.P. (2021). Retention of natural organic matter by ultrafiltration and the mitigation of membrane fouling through pre-treatment, membrane enhancement, and cleaning—A review. J. Water Process Eng..

[5] Wang H., Park M., Liang H., Wu S., Lopez I.J., Ji W., Li G., Snyder S.A. (2017). Reducing ultrafiltration membrane fouling during potable water reuse using pre-ozonation. Water Res..

[6] Zheng X., Khan M.T., Croué J.-P. (2014). Contribution of effluent organic matter (EfOM) to ultrafiltration (UF) membrane fouling: Isolation, characterization, and fouling effect of EfOM fractions. Water Res..

[7] Wang Z., Wan Y., Xie P., Zhou A., Ding J., Wang J., Zhang L., Wang S., Zhang T.C. (2019). Ultraviolet/persulfate (UV/PS) pretreatment of typical natural organic matter (NOM): Variation of characteristics and control of membrane fouling. Chemosphere.

[8] Gao W., Liang H., Ma J., Han M., Chen Z.-l., Han Z.-s., Li G.-b. (2011). Membrane fouling control in ultrafiltration technology for drinking water production: A review. Desalination.

[9] Mao R., Wang Y., Zhao Y., Gao B., Dong M. (2013). Impact of various coagulation technologies on membrane fouling in coagulation/ultrafiltration process. Chem. Eng. J..

[10] Yu W., Graham N.J.D., Fowler G.D. (2016). Coagulation and oxidation for controlling ultrafiltration membrane fouling in drinking water treatment: Application of ozone at low dose in submerged membrane tank. Water Res..

[11] Xing J., Wang H., Cheng X., Tang X., Luo X., Wang J., Wang T., Li G., Liang H. (2018). Application of low-dosage UV/chlorine pre-oxidation for mitigating ultrafiltration (UF) membrane fouling in natural surface water treatment. Chem. Eng. J..

[12] Liu B., Wang M., Yang K., Li G., Shi Z. (2022). Alleviation of Ultrafiltration Membrane Fouling by ClO_2_ Pre-Oxidation: Fouling Mechanism and Interface Characteristics. Membranes.

[13] Cheng X., Feng Y., Li P., Chen K., Yang T., Jin Y., Luo X., Wu D. (2026). Far-UVC photolysis of chlorine to mitigate ultrafiltration membrane fouling: Unraveling the molecular transformation mechanism of natural organic matter. Water Res..

[14] Yang J., Liu Y., Zhao J., Wang H., Li G., Liang H. (2023). Controlling ultrafiltration membrane fouling in surface water treatment via combined pretreatment of O_3_ and PAC: Mechanism investigation on impacts of technological sequence. Sci. Total Environ..

[15] Chen S., Zhao Z., Wang C., Cui F. (2024). VUV coupled with low-dose H_2_O_2_ as pretreatment prior to UF: Performance, mechanisms, DBPs formation and toxicity evaluation. J. Hazard. Mater..

[16] Xu T., Jiang M., Mo S., Wang X., Ren T., Liu Z., Wang Z., Qiu Y., Gu L., Wang X. (2025). Mn−Fe Dual-Metal Assemblages on Carbon-Coated Al_2_O_3_ Spheres for Catalytic Ozonation Oxidation: Structure, Performance, and Reaction Mechanism. ChemSusChem.

[17] Xu T., Jiang M., Mao X., Sher F., Ren T., Xin K., Hussain I., Hua H., Wang X. (2025). Design of high-performance ozone catalysts by identifying and modulating anchoring sites for metal on γ-Al_2_O_3_ spheres. Appl. Catal. B Environ. Energy.

[18] Kasprzyk-Hordern B., Ziółek M., Nawrocki J. (2003). Catalytic ozonation and methods of enhancing molecular ozone reactions in water treatment. Appl. Catal. B Environ..

[19] Zhuo Q., Lu J., Niu J., Crittenden J.C., Yu G., Wang S., Yang B., Chen Z. (2022). Electrocatalytic Oxidation Processes for Treatment of Halogenated Organic Pollutants in Aqueous Solution: A Critical Review. ACS Es&T Eng..

[20] Yun E.-T., Shim J., Choi J., Oh H., Lee J.-H., Fortner J.D., Cho K.H., Lee J. (2026). Advancing membrane fouling control via electrically driven microbubble generation: Phosphidation-enhanced electrocatalytic activity of Ni foam and numerical optimization of operational parameters. Water Res..

[21] Qu F., Liang H., Zhou J., Nan J., Shao S., Zhang J., Li G. (2014). Ultrafiltration membrane fouling caused by extracellular organic matter (EOM) from Microcystis aeruginosa: Effects of membrane pore size and surface hydrophobicity. J. Membr. Sci..

[22] Zeng W., Bai L., Liang H., Li G., Zhang H., Wang J., Gan Z., Lin D., Guo Y., Shao P. (2022). Dual role of boron-doped diamond (BDD) anode in effluent organic matter degradation and ultrafiltration membrane fouling mitigation. Chemosphere.

