# Supplementary material for: Electrocatalytic Oxidation as an Effective Pretreatment for Ultrafiltration Fouling Control: Modulation of EfOM Characteristics and Fouling Pathways
Source: Membranes (Basel). 2026 Sep 25;16(10):317. doi: 10.3390/membranes16100317 (PMC13707238; doi:10.3390/membranes16100317)
Supplement: Supplementary file 1 [file membranes-16-00317-s001.zip › membranes-4525921-supplementary.pdf]

Supplementary Materials

Electrocatalytic Oxidation as an Effective Pretreatment for Ultrafiltration Fouling Control: Modulation of EfOM Characteristics and Fouling Pathways

Tao Xu

PipeChina Institute of Science and Technology, Tianjin 300450, China; xutao62@pipechina.com.cn

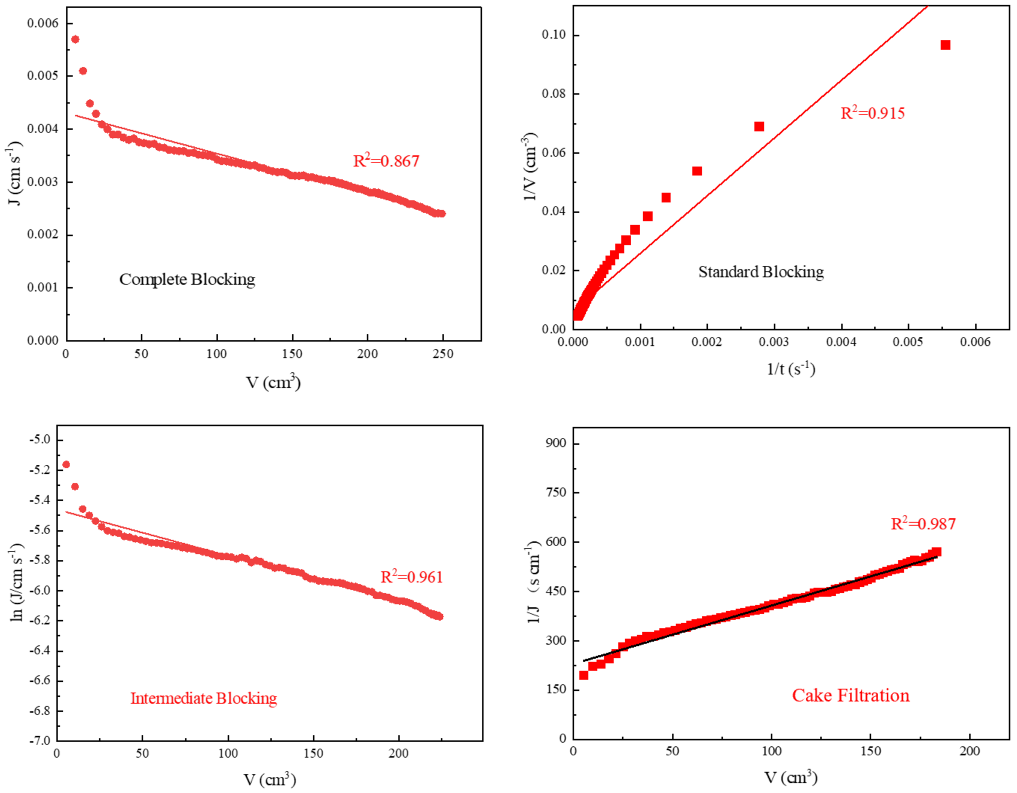

Figure S1. Regression analyses of pristine membrane fouling

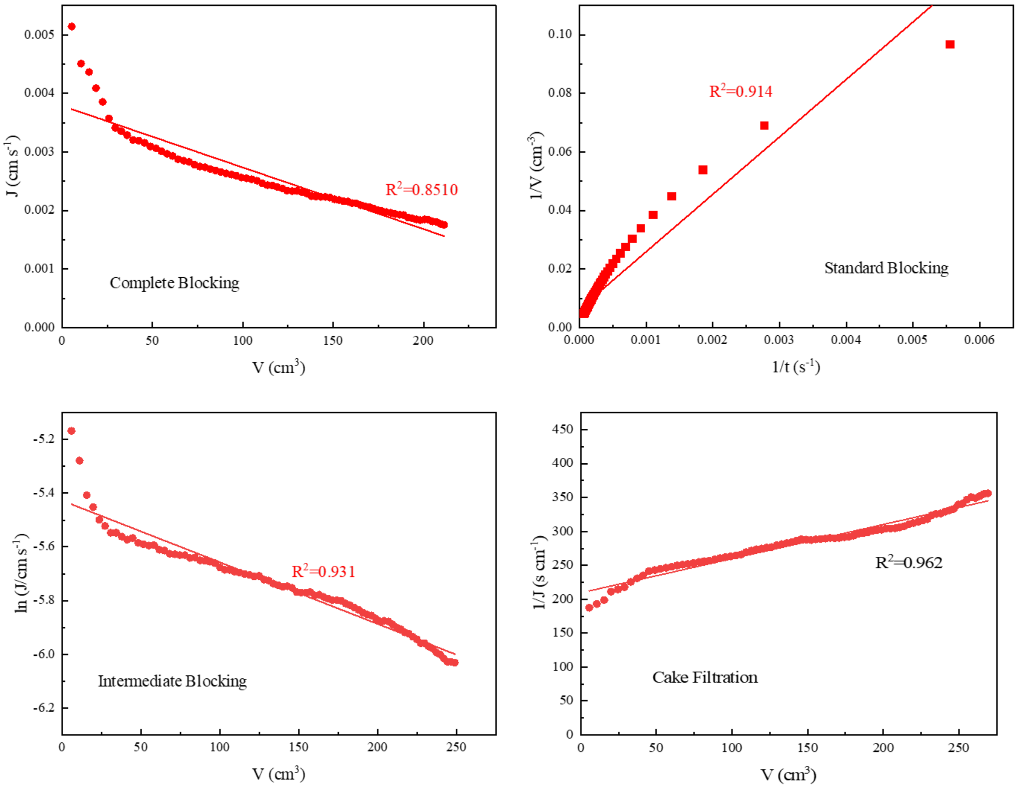

Figure S2. Regression analyses of membrane fouling in the UF + EO (20mA/cm^2) process

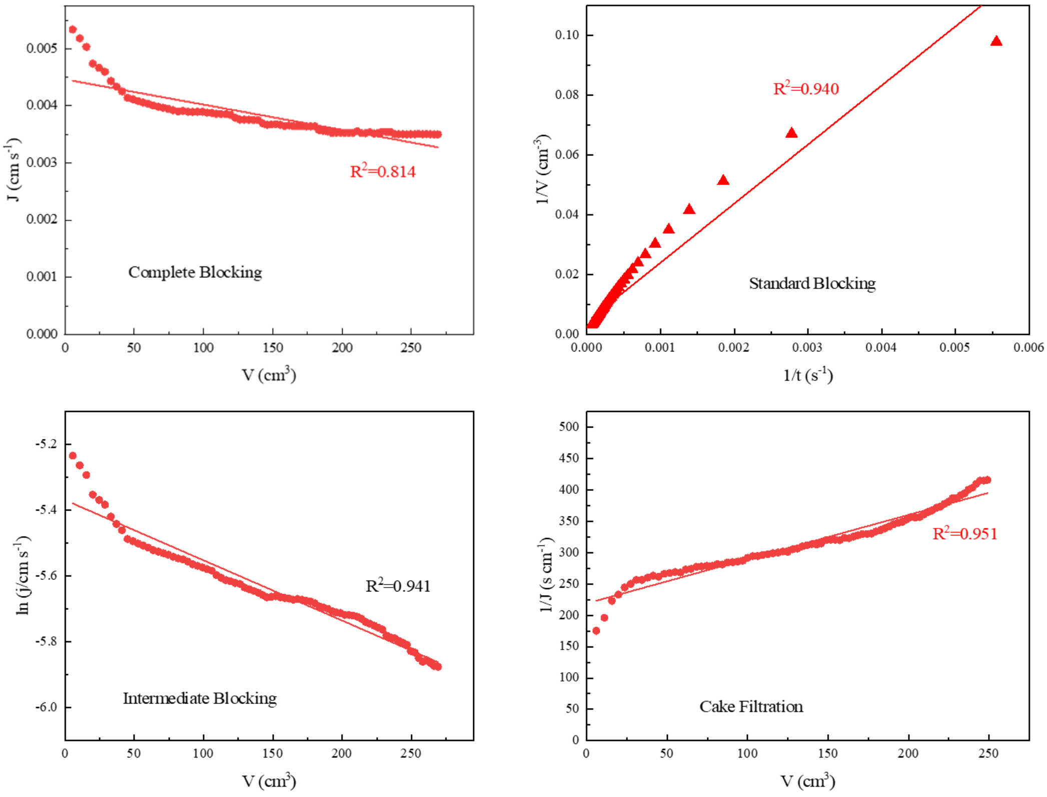

Figure S3. Regression analyses of membrane fouling in the UF + EO (50mA/cm^2) process

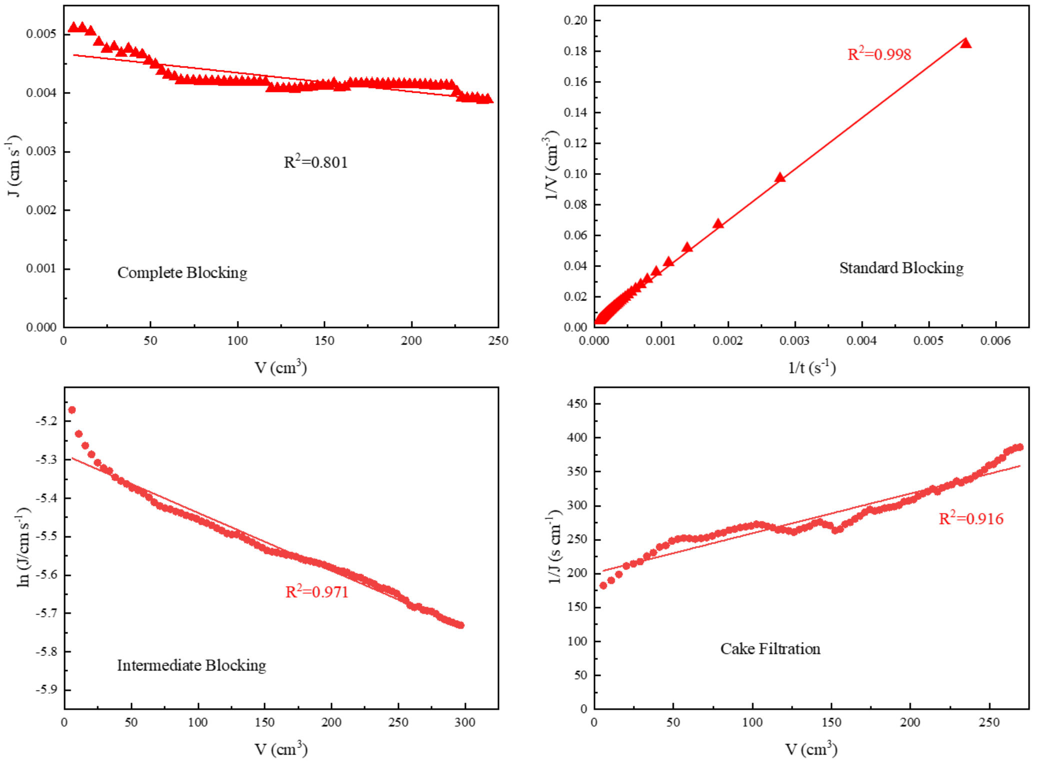

Figure S4. Regression analyses of membrane fouling in UF + EO (70mA/cm^2) process

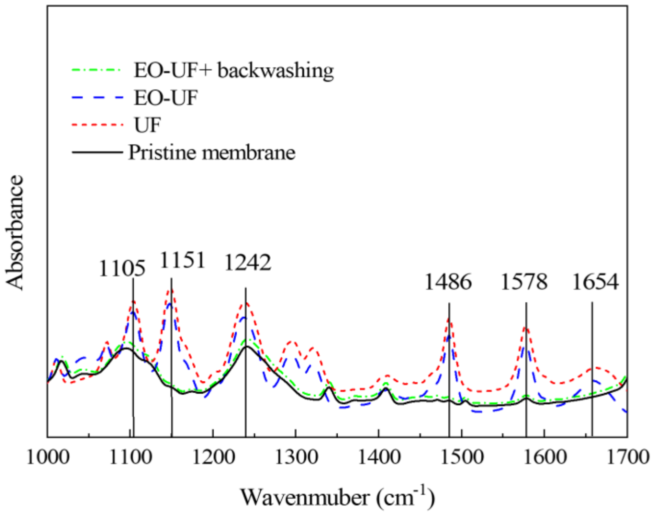

Figure S5. FT-IR spectra of the membrane at different conditions.

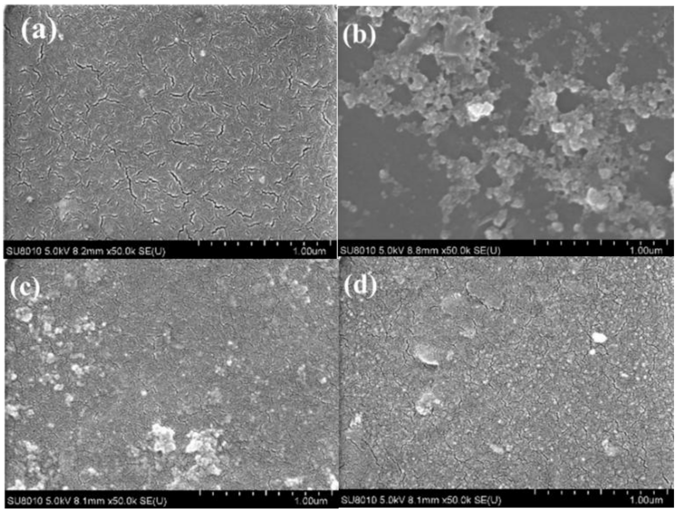

Figure S6. SEM images of membrane surface morphology under different conditions: (a) pristine membrane; (b) membrane after direct UF filtration; (c) membrane after EO-UF filtration; and (d) membrane after EO-UF filtration followed by hydraulic backwashing.

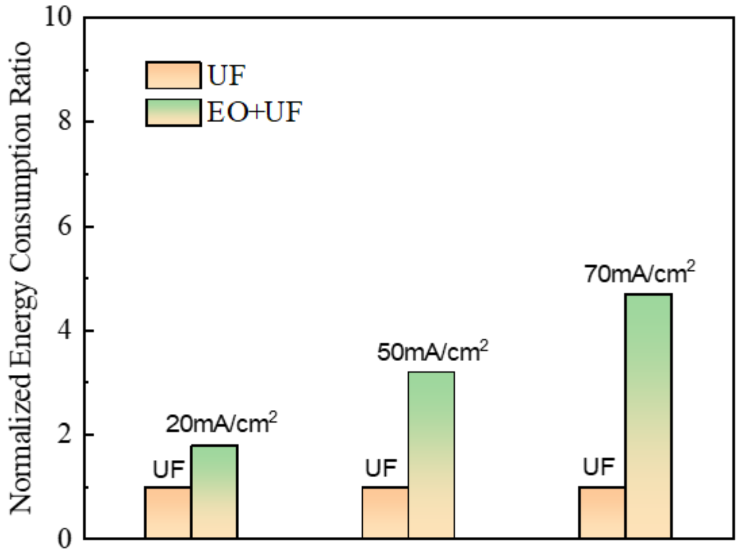

Figure S7. Normalized energy consumption ratio of different systems.
